# Comparison of outcomes of second-line durvalumab plus tremelimumab versus lenvatinib following first-line atezolizumab plus bevacizumab in unresectable hepatocellular carcinoma

**DOI:** 10.1371/journal.pone.0341395

**Published:** 2026-05-07

**Authors:** Chinatsu Nishioka, Yuki Tahata, Kazuki Maesaka, Machiko Kai, Kumiko Shirai, Kazuhiro Murai, Yuki Makino, Yoshinobu Saito, Yasutoshi Nozaki, Tasuku Nakabori, Hisashi Ishida, Takayuki Yakushijin, Sadaharu Iio, Nobuyuki Tatsumi, Kazuho Imanaka, Naruyasu Kakita, Ryotaro Sakamori, Atsushi Hosui, Masanori Miyazaki, Kengo Matsumoto, Masanori Nakahara, Yoshinori Doi, Mitsuru Sakakibara, Hayato Hikita, Takahiro Kodama, Tetsuo Takehara

**Affiliations:** 1 Department of Gastroenterology and Hepatology, The University of Osaka, Graduate School of Medicine, Suita, Osaka, Japan; 2 Department of Gastroenterology and Hepatology, Kansai Rosai Hospital, Amagasaki, Hyogo, Japan; 3 Department of Hepatobiliary and Pancreatic Oncology, Osaka International Cancer Institute, Osaka, Osaka, Japan; 4 Department of Gastroenterology and Hepatology, Ikeda Municipal Hospital, Ikeda, Osaka, Japan; 5 Department of Gastroenterology and Hepatology, Osaka General Medical Center, Osaka, Osaka, Japan; 6 Department of Gastroenterology and Hepatology, Hyogo Prefectural Nishinomiya Hospital, Nishinomiya, Hyogo, Japan; 7 Department of Gastroenterology and Hepatology, Japan Community Healthcare Organization Osaka Hospital, Osaka, Osaka, Japan; 8 Department of Gastroenterology and Hepatology, Itami City Hospital, Itami, Hyogo, Japan; 9 Department of Gastroenterology and Hepatology, Kaizuka City Hospital, Kaizuka, Osaka, Japan; 10 Department of Gastroenterology and Hepatology, National Hospital Organization Osaka National Hospital, Osaka, Osaka, Japan; 11 Department of Gastroenterology and Hepatology, Osaka Rosai Hospital, Sakai, Osaka, Japan; 12 Department of Gastroenterology and Hepatology, Osaka Keisatsu Hospital, Osaka, Osaka, Japan; 13 Department of Gastroenterology and Hepatology, Toyonaka Municipal Hospital, Toyonaka, Osaka, Japan; 14 Department of Gastroenterology and Hepatology, Minoh City Hospital, Minoh, Osaka, Japan; 15 Department of Gastroenterology and Hepatology, Otemae Hospital, Osaka, Osaka, Japan; 16 Department of Gastroenterology and Hepatology, Yao Municipal Hospital, Yao, Osaka, Japan; PearlsInMires, KOREA, REPUBLIC OF

## Abstract

**Background and Aim:**

The optimal second-line therapy following first-line atezolizumab plus bevacizumab remains unascertained. In this study, we compared second-line durvalumab plus tremelimumab with lenvatinib after first-line atezolizumab plus bevacizumab in patients with unresectable hepatocellular carcinoma (uHCC).

**Methods:**

In this retrospective, open-label, non-randomized comparative study, we analyzed real-world data derived from a prospectively registered observational cohort of patients with uHCC who received durvalumab plus tremelimumab (the Dur/Tre group, n = 14) or lenvatinib (the Len group, n = 67) as second-line therapy after first-line atezolizumab plus bevacizumab. Tumor response was assessed using the RECIST criteria version 1.1. Progression-free survival (PFS), overall survival (OS), adverse events (AEs), and changes in the albumin–bilirubin (ALBI) score were compared between the groups.

**Results:**

The objective response and disease control rates were 7.7% and 15.4% in the Dur/Tre group and 23.3% and 76.7% in the Len group, respectively. The median PFS was 1.7 vs. 4.2 months (p < 0.001) and median OS was 5.3 vs. 14.0 months (p = 0.047) in the Dur/Tre and Len groups, both significantly favoring lenvatinib. Multivariable analysis showed that lenvatinib treatment was an independent predictor of longer PFS, and a neutrophil-to-lymphocyte ratio ≥ 3 was an independent predictor of worse OS. Grade ≥ 3 AEs were more frequent with lenvatinib than with durvalumab plus tremelimumab (70.1% vs. 21.4%; p = 0.002). At week 4, the ALBI score was maintained with durvalumab plus tremelimumab (−2.30 to −2.21; p = 0.318) but worsened with lenvatinib (−2.36 to −1.97; p < 0.001).

**Conclusions:**

After first-line atezolizumab plus bevacizumab, second-line lenvatinib achieved superior disease control and longer survival than durvalumab plus tremelimumab. However, grade ≥ 3 AEs were more frequent with lenvatinib than with durvalumab plus tremelimumab. Careful AE management is therefore important when selecting therapy for individual patients.

## Introduction

Systemic therapy for unresectable hepatocellular carcinoma (HCC) commenced with sorafenib, which was approved as a first-line standard of care in 2009 [1,2]. Subsequently, based on the REFLECT trial, lenvatinib was approved for HCC treatment in 2018 [3]. In 2020, the IMbrave150 trial established atezolizumab plus bevacizumab as the initial first-line regimen to incorporate an immune checkpoint inhibitor (ICI) [4]. Thereafter, in 2022, following the HIMALAYA trial, durvalumab plus tremelimumab—the first dual-immunotherapy regimen—was approved for HCC, which further expanded therapeutic options [5]. Contemporary guidelines recommend atezolizumab plus bevacizumab or durvalumab plus tremelimumab as first-line therapy for patients who are eligible for ICI therapy [6,7]. Recently, in 2025, the combination of nivolumab plus ipilimumab demonstrated a significant overall survival (OS) benefit, compared with lenvatinib or sorafenib [8]. Thus, further improvements in outcomes for patients with unresectable HCC are anticipated.

Atezolizumab plus bevacizumab is an established first-line regimen for unresectable HCC [9], with an objective response rate (ORR) of 22.0–28.2% and a disease control rate (DCR) of 69.6–70.6% [10,11]. However, most patients with HCC experience disease progression during treatment with atezolizumab plus bevacizumab and transition to subsequent therapy, and the optimal sequence of systemic treatments thereafter remains unclear [12]. In this regard, real-world studies, including a recent comparison of first-line lenvatinib and second-line lenvatinib after atezolizumab plus bevacizumab, have reported stable tumor responses and favorable survival outcomes with second-line lenvatinib after atezolizumab plus bevacizumab [13–16]. Therefore, lenvatinib may be a useful clinical option after treatment with atezolizumab plus bevacizumab. Additionally, recent real-world studies have suggested the potential utility of durvalumab plus tremelimumab as second-line therapy after first-line atezolizumab plus bevacizumab. In these reports, the DCR ranged from 54.5% to 62.5% [17,18], and anti-CTLA-4-containing therapy was suggested to effectively induce T-cell priming, regardless of prior exposure to atezolizumab plus bevacizumab [17]. Furthermore, after the initiation of durvalumab plus tremelimumab, no significant worsening of the ALBI score was observed at 6 months, at the end of durvalumab plus tremelimumab treatment, or at the end of observation [18]. These findings suggest that durvalumab plus tremelimumab may be a useful treatment option after atezolizumab plus bevacizumab.

In Japan, multiple ICI-based regimens have been approved for HCC, and both durvalumab plus tremelimumab and lenvatinib are recommended as second-line treatment options after first-line atezolizumab plus bevacizumab [19]. Therefore, a comparison of durvalumab plus tremelimumab and lenvatinib as second-line therapy after first-line atezolizumab plus bevacizumab is clinically important; however, direct head-to-head real-world evidence in this setting remains scarce. In this study, we evaluated the efficacy and safety of second-line durvalumab plus tremelimumab after first-line atezolizumab plus bevacizumab and compared these results with those of second-line lenvatinib after first-line atezolizumab plus bevacizumab.

## Methods

### Study design

This multicenter, registry-based, retrospective, open-label, non-randomized comparative study was performed at The University of Osaka Hospital and 18 affiliated institutions of the Osaka Liver Forum. The study used real-world data derived from a prospectively registered observational cohort of patients who received systemic therapy for HCC in routine clinical practice at the participating centers. No formal a priori sample size calculation was performed because this was an exploratory study based on real-world data. All eligible patients identified at the participating centers during the predefined study periods were included in the analysis. We compared the outcomes in patients who received durvalumab plus tremelimumab as second-line therapy after first-line atezolizumab plus bevacizumab (the Dur/Tre group) with those of patients who received lenvatinib as second-line therapy after first-line atezolizumab plus bevacizumab (the Len group).

The study protocol was approved by The University of Osaka Hospital Ethical Review Board and the ethics committees of all participating institutions (UMIN000034611). Written informed consent was obtained from all participants at each participating institution. The data were accessed for research purposes between November 16, 2024, and September 3, 2025. After data collection, the authors did not have access to information that could identify individual participants.

### Study population and enrollment period

In the Dur/Tre group, first-line atezolizumab plus bevacizumab treatment was initiated between October 2020 and January 2024, and second-line durvalumab plus tremelimumab was started between April 2023 and June 2024. In routine clinical practice, the decision to initiate second-line durvalumab plus tremelimumab was made after the attending physicians comprehensively assessed the general condition, comorbidities, and prior tolerance to atezolizumab plus bevacizumab for each patient, and determined that the patient would be able to tolerate ICI rechallenge. The Len group included 67 patients who received lenvatinib following first-line atezolizumab plus bevacizumab between October 2020 and January 2023, as reported previously [13]. This cohort was derived from the period before durvalumab plus tremelimumab became available and served as a historical control. Lenvatinib was administered when the attending physicians deemed that the patient would be likely to tolerate lenvatinib after atezolizumab plus bevacizumab. Treatment-selection bias between lenvatinib and durvalumab plus tremelimumab after atezolizumab plus bevacizumab was considered to be minimized by using this historical cohort, because all patients in the Len group had been treated before durvalumab plus tremelimumab treatment became available. Accordingly, patients with unresectable HCC who received durvalumab plus tremelimumab or lenvatinib as second-line treatment following first-line atezolizumab plus bevacizumab were selected.

The inclusion criteria for this study were as follows: (1) patients with unresectable HCC who received durvalumab plus tremelimumab or lenvatinib as second-line therapy after first-line atezolizumab plus bevacizumab at hospitals participating in the Osaka Liver Forum; (2) patients with an Eastern Cooperative Oncology Group performance status of 0 or 1; and (3) patients who provided written informed consent for participation in the prospective observational cohort.

The exclusion criteria were as follows: (1) inability to receive contrast-enhanced imaging for tumor response evaluation; (2) initiation of second-line durvalumab plus tremelimumab or lenvatinib >3 months after discontinuation of atezolizumab plus bevacizumab; (3) participation in a clinical trial; and (4) observation period of < 4 weeks.

The final analytic cohort comprised 14 patients in the Dur/Tre group and 67 patients in the Len group after application of the predefined eligibility and exclusion criteria.

### Lenvatinib administration

Lenvatinib was administered orally once per day. According to the approved dosage in Japan [3,20,21], the initial dose was determined by body weight: 12 and 8 mg/day for patients ≥ 60 and < 60 kg, respectively. Dose reductions or interruptions were allowed based on the severity of adverse events (AEs), in accordance with the prescribing recommendations.

### Durvalumab plus tremelimumab administration

Durvalumab plus tremelimumab was administered according to the previously described clinical trial protocol [5] as follows: 300 mg tremelimumab and 1500 mg durvalumab administered on day 1, followed by 1500 mg durvalumab every 4 weeks. Dose interruptions were permitted based on the severity of AEs, in accordance with the prescribing recommendations.

### Outcome measures

The primary outcomes of the study were progression-free survival (PFS) and OS. The secondary outcomes were ORR, DCR, the incidence of treatment-related AEs, changes in the albumin–bilirubin (ALBI) score, and subsequent therapy. ORR was defined as the proportion of evaluable patients who achieved complete response (CR) or partial response (PR) as their best overall response. DCR was defined as the proportion of evaluable patients who achieved CR, PR, or stable disease (SD) as their best overall response.

### Treatment evaluation

Based on the contrast-enhanced computed tomography or magnetic resonance imaging findings, the tumor response was assessed using the Response Evaluation Criteria in Solid Tumors (RECIST) version 1.1. CR was defined as the disappearance of all target lesions, and PR as at least a 30% decrease in the sum of the diameters of target lesions from baseline. Progressive disease (PD) was defined as at least a 20% increase in the sum of the diameters of target lesions relative to the smallest sum recorded during treatment, with an absolute increase of at least 5 mm, or the appearance of one or more new lesions or unequivocal progression of non-target lesions. SD was defined as neither sufficient shrinkage to qualify for PR nor sufficient increase to qualify for PD [22]. Imaging evaluations were performed at baseline, weeks 4 and 8 after treatment initiation, and every 8 weeks thereafter. AEs were evaluated and graded according to the Common Terminology Criteria for Adverse Events (CTCAE), version 5.0. After disease progression, switching to another treatment regimen was allowed at the discretion of the attending physicians.

### Statistical analysis

Clinical parameters are presented as counts and percentages for categorical variables, and as medians and ranges for continuous variables. Categorical variables were compared using the chi-square test or Fisher’s exact test, and continuous variables were compared with the Mann–Whitney *U* test. The PFS and OS were estimated using the Kaplan–Meier method, and differences between groups were assessed using the log-rank test. Independent prognostic factors associated with PFS and OS were identified using the Cox proportional hazards model. Based on previous studies [23–27], the following covariates were prespecified for inclusion in the multivariable analysis: treatment regimen, age, sex, etiology, Barcelona Clinic Liver Cancer stage, modified ALBI grades, serum α-fetoprotein level, des-γ-carboxy prothrombin (DCP) level, neutrophil-to-lymphocyte ratio (NLR), duration of prior atezolizumab plus bevacizumab and best response to prior atezolizumab plus bevacizumab treatment. Changes in the ALBI score within each group were compared using the Wilcoxon signed-rank test. *p* < 0.05 was considered statistically significant. All statistical analyses were conducted using SPSS statistical software (version 29.0) for Windows (IBM Corp., Armonk, NY, USA).

## Results

### Baseline characteristics of patients treated with second-line durvalumab plus tremelimumab versus lenvatinib after first-line atezolizumab plus bevacizumab

Initially, 35 patients who received durvalumab plus tremelimumab immediately after atezolizumab plus bevacizumab were screened for inclusion in the Dur/Tre group. After applying the exclusion criteria, 14 patients who had received first-line atezolizumab plus bevacizumab followed by second-line durvalumab plus tremelimumab were included in the analysis. The Len group comprised 67 patients who received second-line lenvatinib after first-line atezolizumab plus bevacizumab (**S1 Fig**). The median age was 73 and 75 years (p = 0.797), and male accounted for 64.3% and 83.6% of the patients in the Dur/Tre and Len groups, respectively (p = 0.137). Child–Pugh class A was observed in 85.7% and 86.6% of the patients in the Dur/Tre and Len groups, respectively (p = 1.000). In the Dur/Tre and Len groups, 64.3% and 68.7% of the patients exhibited five or more intrahepatic tumors (p = 0.760), 35.7% and 31.3% had macrovascular invasion (p = 0.760), and 42.9% and 47.8% had extrahepatic metastasis (p = 0.777), respectively. First-line atezolizumab plus bevacizumab had been administered for ≥ 3 months in 57.1% and 61.2% of the patients (p = 0.773). Additionally, a total of 64.3% and 53.7% of patients in the Dur/Tre and Len groups, respectively, had a best response of CR, PR, or SD to prior atezolizumab plus bevacizumab treatment (p = 0.562). All patients in the Dur/Tre group discontinued prior atezolizumab plus bevacizumab because of disease progression, whereas 89.6% patients in the Len group discontinued prior atezolizumab plus bevacizumab because of disease progression and 10.4% because of adverse events (p = 0.345). Overall, no significant between-group differences were observed in baseline characteristics (**Table 1**).

**Table 1:** Patient Characteristics.

Characteristic		Dur/Tre group (n = 14)	Len group (n = 67)	p value
Age, years	Median (range)	73 (31–88)	75 (48–90)	0.797
Sex, n (%)	MaleFemale	9 (64.3)5 (35.7)	56 (83.6)11 (16.4)	0.137
ECOG PS, n (%)	0 1	12 (85.7)2 (14.3)	63 (94.0)4 (6.0)	0.276
Etiology, n (%)	ViralNon-viral	4 (28.6)10 (71.4)	32 (47.8)35 (52.2)	0.244
Maximum intrahepatic tumor size, mm	Median (range)	37 (12–120)	33 (0–168)	0.546
Intrahepatic tumor number, n (%)	≤ 4 ≥ 5	5 (35.7)9 (64.3)	21 (31.3)46 (68.7)	0.760
Macrovascular invasion, n (%)	AbsentPresent	9 (64.3)5 (35.7)	46 (68.7)21 (31.3)	0.760
Extrahepatic metastasis, n (%)	AbsentPresent	8 (57.1) 6 (42.9)	35 (52.2)32 (47.8)	0.777
BCLC stage, n (%)	A or B C	5 (35.7)9 (64.3)	24 (35.8) 43 (64.2)	1.000
Child-Pugh class, n (%)	A B	12 (85.7)2 (14.3)	58 (86.6)9 (13.4)	1.000
mALBI grade, n (%)	1 or 2a2b or 3	7 (50.0)7 (50.0)	38 (56.7)29 (43.3)	0.770
AFP, ng/ml, n (%)	< 400 ≥ 400	9 (64.3) 5 (35.7)	48 (71.6) 19 (28.4)	0.748
DCP, mAU/ml, n (%)	< 400 ≥ 400	6 (42.9) 8 (57.1)	17 (26.2) 48 (73.8)	0.330
NLR, n (%)	< 3 ≥ 3	4 (28.6) 10 (71.4)	32 (49.2) 33 (50.8)	0.238
Duration of prior Atezo/Beva, months	< 3 ≥ 3	6 (42.9) 8 (57.1)	26 (38.8) 41 (61.2)	0.773
Best response to prior Atezo/Beva	CR/PR/SD PD	9 (64.3) 5 (35.7)	36 (53.7) 31 (46.3)	0.562
Discontinuation reason of prior Atezo/Beva	PD AE	14 (100.0) 0 (0.0)	60 (89.6) 7 (10.4)	0.345

Abbreviations: Dur/Tre, durvalumab plus tremelimumab; Len, lenvatinib; ECOG PS, Eastern Cooperative Oncology Group performance status; BCLC, Barcelona Clinic Liver Cancer; mALBI, modified albumin-bilirubin; AFP, α-fetoprotein; DCP, des-gamma-carboxy prothrombin; NLR, neutrophil-to-lymphocyte ratio; Atezo/Beva, atezolizumab plus bevacizumab; CR, complete response; PR, partial response; SD, stable disease; PD, progressive disease; AE, adverse event.

The median observation periods in the Dur/Tre and Len groups were 5.6 months (1.9–15.0 months) and 8.6 months (1.5–23.9 months), respectively.

### Comparative efficacy and safety of second-line durvalumab plus tremelimumab versus lenvatinib after first-line atezolizumab plus bevacizumab

According to the best therapeutic response assessed using RECIST version 1.1, 0 (0%), 1 (7.7%), 1 (7.7%), and 11 (84.6%) patients in the Dur/Tre group exhibited a CR, PR, SD, and PD, respectively, while the response was not evaluable (NE) in 1 patient, yielding an ORR of 7.7% and a DCR of 15.4%. In the Len group, 1 (1.7%), 13 (21.7%), 32 (53.3%), and 14 (23.3%) patients showed a CR, PR, SD, and PD, respectively, while 7 had NE responses, yielding an ORR of 23.3% and a DCR of 76.7%. The ORR did not differ significantly between the two groups (p = 0.279), whereas the DCR was significantly higher in the Len group than in the Dur/Tre group (p < 0.001) (**Table 2**).

**Table 2:** Best therapeutic response.

	RECIST (version 1.1)		
	Dur/Tre group	Len group	p value
CR	0 (0%)	1 (1.7%)	–
PR	1 (7.7%)	13 (21.7%)	–
SD	1 (7.7%)	32 (53.3%)	–
PD	11 (84.6%)	14 (23.3%)	–
ORR	1 (7.7%)	14 (23.3%)	0.279
DCR	2 (15.4%)	46 (76.7%)	< 0.001
NE	1	7	

Abbreviations: RECIST, Response Evaluation Criteria in Solid Tumors; Dur/Tre, durvalumab plus tremelimumab; Len, lenvatinib; CR, complete response; PR, partial response; SD, stable disease; PD, progressive disease; ORR, objective response rate; DCR, disease control rate; NE, not evaluable.

**Fig 1:**
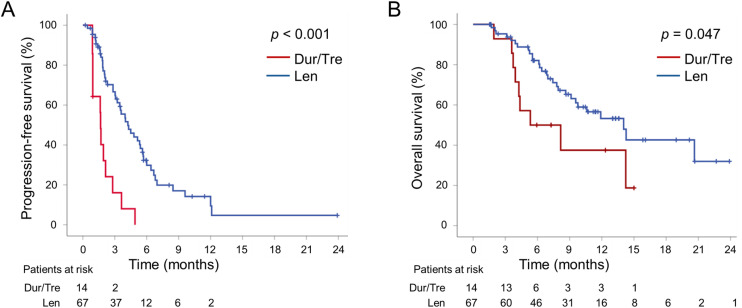
Comparisons of PFS (A) and OS (B) between the Dur/Tre and Len groups. Abbreviations: PFS, progression-free survival; OS, overall survival; Dur/Tre, durvalumab plus tremelimumab; Len, lenvatinib.

The median PFS was 1.7 (95% CI, 1.6–1.8) and 4.2 (95% CI, 2.9–5.6) months in the Dur/Tre and Len groups, respectively, and favored lenvatinib (p < 0.001). The median OS was 5.3 (95% CI, 0.3–10.4) and 14.0 (95% CI, 9.8–18.2) months in the Dur/Tre and Len groups, respectively, favoring lenvatinib (p = 0.047; **Fig 1A and 1B**).

The incidence of any-grade and grade ≥ 3 AEs was significantly higher in the Len group than in the Dur/Tre group (any grade: 98.5% vs. 78.6%, p = 0.015; grade ≥ 3: 70.1% vs. 21.4%, p = 0.002) (**Table 3**). Among any-grade AEs, fatigue (56.7% vs. 7.1%, p < 0.001), proteinuria (55.2% vs. 7.1%, p < 0.001), hypertension (47.8% vs. 0%, p < 0.001), decreased appetite (52.2% vs. 7.1%, p = 0.002), thrombocytopenia (25.4% vs. 0%, p = 0.034) and hand-foot skin reaction (29.9% vs. 0%, p = 0.017) were significantly more common in the Len group than in the Dur/Tre group. Similarly, among AEs with grade ≥ 3, proteinuria was significantly more frequent in the Len group than in the Dur/Tre group (29.9% vs. 0%, p = 0.017). Treatment discontinuation due to AEs occurred in 2 patients (14.3%) in the Dur/Tre group and 18 patients (26.9%) in the Len group, with no significant difference between the two groups (p = 0.499). In the Dur/Tre group, systemic corticosteroids were administered for immune-related adverse events (irAEs) in 2 patients (14.3%)—rash in 1 (7.1%) and colitis in 1 (7.1%).

**Table 3:** Adverse Events.

	Any-grade; n (%)				≥ Grade 3; n (%)		
	Dur/Tre group	Len group	p value		Dur/Tre group	Len group	p value
Any adverse event	11 (78.6)	66 (98.5)	0.015		3 (21.4)	47 (70.1)	0.002
Rash	4 (28.6)	10 (14.9)	0.249		1 (7.1)	2 (3.0)	0.439
Increased AST or ALT	3 (21.4)	12 (17.9)	0.716		0 (0.0)	3 (4.5)	1.000
Hypothyroidism	2 (14.3)	25 (37.3)	0.125		0 (0.0)	0 (0.0)	NA
Colitis/diarrhea	1 (7.1)	10 (14.9)	0.679		0 (0.0)	1 (1.5)	1.000
Adrenal insufficiency	1 (7.1)	0 (0.0)	0.173		0 (0.0)	0 (0.0)	NA
Fatigue	1 (7.1)	38 (56.7)	< 0.001		0 (0.0)	8 (11.9)	0.339
Proteinuria	1 (7.1)	37 (55.2)	< 0.001		0 (0.0)	20 (29.9)	0.017
Decreased appetite	1 (7.1)	35 (52.2)	0.002		0 (0.0)	6 (9.0)	0.583
Hypertension	0 (0.0)	32 (47.8)	< 0.001		0 (0.0)	9 (13.4)	0.347
Thrombocytopenia	0 (0.0)	17 (25.4)	0.034		0 (0.0)	3 (4.5)	1.000
Hand-foot-skin reaction	0 (0.0)	20 (29.9)	0.017		0 (0.0)	2 (3.0)	1.000
Liver failure	1 (7.1)	0 (0.0)	0.173		1 (7.1)	0 (0.0)	0.173
Bleeding	1 (7.1)	3 (4.5)	0.539		1 (7.1)	1 (1.5)	0.318

Abbreviations: Dur/Tre, durvalumab plus tremelimumab; Len, lenvatinib; AST, aspartate aminotransferase; ALT, alanine aminotransferase; NA, not applicable.

### Clinical factors associated with PFS and OS in the overall cohort

The pretreatment clinical variables were analyzed to identify risk factors associated with shorter PFS and OS in the overall cohort. In the multivariable analysis including treatment regimen, only lenvatinib treatment (HR 0.271, 95% CI 0.129–0.567; *p* < 0.001) was identified as an independent factor associated with prolonged PFS (**Table 4**). In the multivariable analysis using the same covariates as that for PFS, only elevated NLR (≥ 3; HR 2.394, 95% CI 1.067–5.375; *p* = 0.034) was independently associated with OS (**Table 5**). Median OS was shorter in patients with NLR ≥ 3 than in those with NLR < 3 (8.6 vs. 20.6 months; **S2 Fig**).

**Table 4:** Multivariable analyses of PFS-related factors.

Variables	Category	Multivariable analysis	p value
		Hazard ratio^†^ (95% CI)	
Treatment	Dur/Tre Len	1 0.271 (0.129–0.567)	< 0.001
Age, years	< 75 ≥ 75	1 1.469 (0.790–2.733)	0.224
Sex	MaleFemale	1 1.356 (0.668–2.750)	0.399
Etiology	ViralNon-viral	1 1.249 (0.663–2.353)	0.492
BCLC stage	A or B C	1 1.635 (0.755–3.543)	0.213
mALBI grade	1 or 2a2b or 3	1 1.133 (0.626–2.050)	0.680
AFP, ng/ml	< 400 ≥ 400	1 1.426 (0.709–2.867)	0.320
DCP, mAU/ml	< 400 ≥ 400	1 1.007 (0.527–1.923)	0.983
NLR	< 3 ≥ 3	1 1.603 (0.865–2.971)	0.134
Duration of prior Atezo/Beva, months	< 3 ≥ 3	1 0.825 (0.395–1.722)	0.608
Best response to prior Atezo/Beva	CR/PR/SD PD	1 0.851 (0.382–1.896)	0.692

Abbreviations: CI, confidence interval; PFS, progression-free survival; Dur/Tre, durvalumab plus tremelimumab; Len, lenvatinib; BCLC, Barcelona Clinic Liver Cancer; mALBI, modified albumin-bilirubin; AFP, α-fetoprotein; DCP, des-gamma-carboxy prothrombin; NLR, neutrophil-to-lymphocyte ratio; Atezo/Beva, atezolizumab plus bevacizumab

† Adjusted for treatment, age, sex, etiology, BCLC stage, mALBI grade, AFP, DCP, NLR, duration of prior Atezo/Beva, best response to prior Atezo/Beva

**Table 5:** Multivariable analyses of OS-related factors.

Variables	Category	Multivariable analysis	p value
		Hazard ratio^†^ (95% CI)	
Treatment	Dur/Tre Len	1 0.431 (0.157–1.184)	0.103
Age, years	< 75 ≥ 75	1 1.311 (0.538–3.196)	0.551
Sex	MaleFemale	1 1.897 (0.726–4.960)	0.192
Etiology	ViralNon-viral	1 1.016 (0.431–2.397)	0.971
BCLC stage	A or B C	1 1.458 (0.425–5.005)	0.549
mALBI grade	1 or 2a2b or 3	1 1.557 (0.741–3.272)	0.242
AFP, ng/ml	< 400 ≥ 400	1 1.165 (0.538–2.525)	0.698
DCP, mAU/ml	< 400 ≥ 400	1 1.943 (0.734–5.139)	0.181
NLR	< 3 ≥ 3	1 2.394 (1.067–5.375)	0.034
Duration of prior Atezo/Beva, months	< 3 ≥ 3	1 0.966 (0.354–2.637)	0.947
Best response to prior Atezo/Beva	CR/PR/SD PD	1 2.480 (0.753–8.168)	0.135

† Adjusted for treatment, age, sex, etiology, BCLC stage, mALBI grade, AFP, DCP, NLR, duration of prior Atezo/Beva, best response to prior Atezo/Beva.

Abbreviations: CI, confidence interval; OS, overall survival; Dur/Tre, durvalumab plus tremelimumab; Len, lenvatinib; BCLC, Barcelona Clinic Liver Cancer; mALBI, modified albumin-bilirubin; AFP, α-fetoprotein; DCP, des-gamma-carboxy prothrombin; NLR, neutrophil-to-lymphocyte ratio; Atezo/Beva, atezolizumab plus bevacizumab.

### Changes in the ALBI score

**Fig 2:**
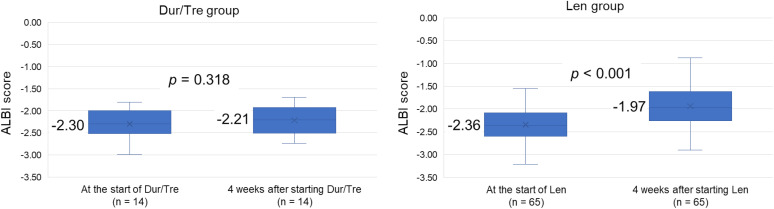
Within-group changes in the ALBI score between treatment initiation and week 4 in the Dur/Tre and Len groups (Dur/Tre, n = 14; Len, n = 65). Two patients in the Len group were excluded because week 4 albumin and total bilirubin data were unavailable. Abbreviations: ALBI, albumin–bilirubin; Dur/Tre, durvalumab plus tremelimumab; Len, lenvatinib..

The median ALBI score in the Dur/Tre group was −2.30 (IQR, −2.51 to −2.00) at treatment initiation and −2.21 (IQR, −2.50 to −1.92) at week 4, without significant deterioration (*p* = 0.318). In the Len group, the median ALBI score was −2.36 (IQR, −2.62 to −2.07) at initiation and worsened to −1.97 (IQR, −2.27 to −1.61) at week 4 (*p* < 0.001; **Fig 2**). Two patients in the Len group were excluded from this analysis because their serum albumin and total bilirubin levels at week 4 after treatment initiation were not measured.

### Subsequent therapy

In the Dur/Tre group, after the discontinuation of durvalumab plus tremelimumab, 10 of the 14 patients (71.4%) received subsequent therapy, including systemic treatment in 9 patients (64.3%) and transcatheter arterial chemoembolization (TACE) in 1 patient (7.1%). The remaining 4 (28.6%) patients received best supportive care. In the Len group, after discontinuation of lenvatinib, 48 of the 66 patients (72.7%) received subsequent therapy, including systemic treatment in 29 patients (43.9%), systemic treatment plus TACE in 3 patients (4.5%), systemic treatment plus hepatic arterial infusion chemotherapy (HAIC) in 1 patient (1.5%), TACE in 5 patients (7.6%), TACE plus continuation beyond progressive disease (PD) in 1 patient (1.5%), transcatheter arterial infusion/HAIC in 2 patients (3.0%), radiation therapy (RT) in 5 patients (7.6%), RT plus continuation beyond PD in 1 patient (1.5%), and continuation beyond PD alone in 1 patient (1.5%). The remaining 18 (27.3%) patients received best supportive care. In addition, 1 patient remained on lenvatinib at the data-cut-off timepoint (**S1 Table**). No significant between-group difference was observed in the proportion of patients that received subsequent therapy (*p* = 1.000).

## Discussion

This multicenter collaborative study of patients with unresectable HCC compared the efficacy and safety of second-line durvalumab plus tremelimumab with lenvatinib following first-line atezolizumab plus bevacizumab. The results showed that the Len group had significantly better PFS, OS (Fig 1A and 1B), and DCR (Table 2) than the Dur/Tre group. However, severe AEs were significantly more frequent in the Len group than in the Dur/Tre group (Table 3). Unlike the Dur/Tre group, the Len group also exhibited a significant worsening of the ALBI score after treatment initiation (Fig 2). To the best of our knowledge, this is the first report of a direct comparison of second-line durvalumab plus tremelimumab and lenvatinib after first-line atezolizumab plus bevacizumab.

On second-line therapy after first-line atezolizumab plus bevacizumab, the Len group showed superior PFS, OS, and DCR compared with the Dur/Tre group. Maesaka et al. recently compared first-line lenvatinib with second-line lenvatinib after atezolizumab plus bevacizumab and reported comparable efficacy between the two groups [13]. Other real-world studies have also reported stable responses and favorable survival with second-line lenvatinib after atezolizumab plus bevacizumab [14–16]; therefore, lenvatinib can be expected to be effective as second-line therapy after atezolizumab plus bevacizumab. Pharmacologically, prolonged programmed death-ligand 1 (PD-L1) blockade on CD8 + T cells induced by atezolizumab plus bevacizumab may potentiate the antitumor activity of subsequent lenvatinib treatment [28,29]. In contrast, several reports suggest reduced effectiveness of durvalumab plus tremelimumab after atezolizumab plus bevacizumab [24,30]. Fujiwara et al. identified an independent association between prior atezolizumab plus bevacizumab and shorter OS (HR 27.38, 95% CI 1.92–391.50; *p* = 0.01) and PFS (HR 6.25, 95% CI 1.16–33.71; *p* = 0.03) in multivariable Cox analyses of patients treated with durvalumab plus tremelimumab [24]. Additionally, Mori et al. reported that, compared with first-line durvalumab plus tremelimumab and later-line durvalumab plus tremelimumab without prior atezolizumab plus bevacizumab, later-line durvalumab plus tremelimumab with prior atezolizumab plus bevacizumab showed significantly worse ORR/DCR and PFS rates [30]. The potential mechanisms underlying the attenuated durvalumab plus tremelimumab efficacy after atezolizumab plus bevacizumab include resistance to programmed death 1 (PD-1)/PD-L1 blockade with T-cell exhaustion [31], diminished T-cell priming owing to anti-vascular endothelial growth factor (VEGF) resistance [32], and rebound VEGF-mediated immunosuppression after bevacizumab [33]. However, recent real-world studies have reported DCRs of 54.5%–62.5% for durvalumab plus tremelimumab administered as second-line therapy after first-line atezolizumab plus bevacizumab, suggesting that this regimen may offer a certain degree of clinical benefit [17,18]. Thus, our findings should be interpreted with caution because the number of patients in the Dur/Tre group was limited in the present study. Moreover, research using large prospective datasets under uniform conditions regarding durvalumab plus tremelimumab after atezolizumab plus bevacizumab remains scarce, warranting further investigation.

In terms of hepatic reserve, the Dur/Tre group maintained the ALBI score from treatment initiation to week 4, whereas the Len group exhibited a worsening ALBI score (Fig 2). Miura et al. demonstrated the preservation of liver function before and after durvalumab plus tremelimumab in patients with HCC who were treated with durvalumab plus tremelimumab following atezolizumab plus bevacizumab [18]. Additionally, Tamaki et al. reported that durvalumab plus tremelimumab can maintain liver function throughout treatment even in later-line settings and in patients with impaired baseline function [34]. Accordingly, durvalumab plus tremelimumab is considered likely to preserve liver function. In our cohort, such preservation likely contributed to a transition rate to subsequent therapy that was comparable to that in the Len group (S1 Table). Furthermore, our observations are consistent with those of previous studies that reported an early decline in liver function in a subset of patients treated with lenvatinib [35–37]. Thus, from the standpoint of preserving liver function, durvalumab plus tremelimumab may be considered a reasonable option even after atezolizumab plus bevacizumab.

Compared to the Dur/Tre group, the incidence of AEs was significantly higher in the Len group, wherein 98.5% patients had at least one AE and 70.1% patients had grade ≥ 3 AEs; fatigue, proteinuria, decreased appetite, and hypertension were common (Table 3). Lenvatinib injures the vascular endothelium, glomerular endothelium/podocytes, and the thyroid capillary network via VEGF/VEGF receptor inhibition, thereby leading to hypertension, proteinuria, and hypothyroidism; furthermore, fatigue and anorexia have a multifactorial pathogenesis [38–40]. The AE profile observed in the Len group in this study was broadly consistent with previous reports of patients treated with lenvatinib after atezolizumab plus bevacizumab [27–29]. Although rash and hepatic dysfunction were relatively frequent in the Dur/Tre group, the overall incidence of AEs—any-grade and grade ≥ 3—was lower than that in the Len group. However, the activation of tumor- and self-reactive T cells by durvalumab plus tremelimumab can systemically injure normal tissues and induce T-cell–mediated irAEs, such as dermatitis and autoimmune hepatitis–like inflammation [41,42]. Accordingly, the AE profile in the Dur/Tre group showed a pattern similar to that reported by Miura et al. for durvalumab plus tremelimumab administered after atezolizumab plus bevacizumab. Nevertheless, the frequency of severe AEs with durvalumab plus tremelimumab after atezolizumab plus bevacizumab was low, which supports an acceptable safety profile.

This study has some limitations. First, the sample size was limited, especially in the Dur/Tre group. Although relevant covariates were adjusted for, the imbalance in sample size between the Dur/Tre and Len groups, together with the wide confidence intervals, limits the clinical applicability of the findings. Second, clinical differences in treatment availability and practice patterns between durvalumab plus tremelimumab and lenvatinib cannot be fully excluded. Therefore, the Len group was constituted from a historical cohort that was treated before durvalumab plus tremelimumab became available to mitigate this real-world bias. Nevertheless, as the patients were not prospectively allocated to either regimen, residual selection bias cannot be ruled out.

In conclusion, lenvatinib showed better treatment outcomes than durvalumab plus tremelimumab as second-line therapy after atezolizumab plus bevacizumab; however, severe AEs were more frequent with lenvatinib. Therefore, careful AE management is essential when selecting second-line therapy in patients with unresectable HCC after atezolizumab plus bevacizumab.

## Supporting information

S1 Fig Flowchart of the participant enrollment. Abbreviations: Dur/Tre, durvalumab plus tremelimumab; Len, lenvatinib; Atezo/Beva, atezolizumab plus bevacizumab; PS, performance status.
(PPTX)

S2 Fig Comparisons of OS between patients with NLR ≥ 3 and <3. Abbreviations: OS, overall survival; NLR, neutrophil-to-lymphocyte ratio.
(PPTX)

S1 Table Subsequent Therapy. Abbreviations: Dur/Tre, durvalumab plus tremelimumab; Len, lenvatinib; TACE, transcatheter arterial chemoembolization; HAIC, hepatic arterial infusion chemotherapy; PD, progressive disease; TAI, transcatheter arterial infusion; RT, radiation therapy.
(DOCX)

S1 Data Raw data used for the analyses. (XLSX)

## Abbreviations

AE: adverse event
ALBI: albumin–bilirubin
Atezo/Beva: atezolizumab plus bevacizumab
CI: confidence interval
CR: complete response
CTCAE: Common Terminology Criteria for Adverse Events
DCR: disease control rate
Dur/Tre: durvalumab plus tremelimumab
HAIC: hepatic arterial infusion chemotherapy
HCC: hepatocellular carcinoma
ICI: immune checkpoint inhibitor
IQR: interquartile range
irAE: immune-related adverse event
Len: lenvatinib
ORR: objective response rate
OS: overall survival
PD: progressive disease
PD-1: programmed death 1
PD-L1: programmed death-ligand 1
PFS: progression-free survival
PR: partial response
RECIST: Response Evaluation Criteria in Solid Tumors
RT: radiation therapy
SD: stable disease
TACE: transcatheter arterial chemoembolization
VEGF: vascular endothelial growth factor
