# Supplementary figures and images for: Comparison of outcomes of second-line durvalumab plus tremelimumab versus lenvatinib following first-line atezolizumab plus bevacizumab in unresectable hepatocellular carcinoma
Source: PLoS One. 2026 May 7;21(5):e0341395. doi: 10.1371/journal.pone.0341395 (PMC13152131; doi:10.1371/journal.pone.0341395)

## Slide 1
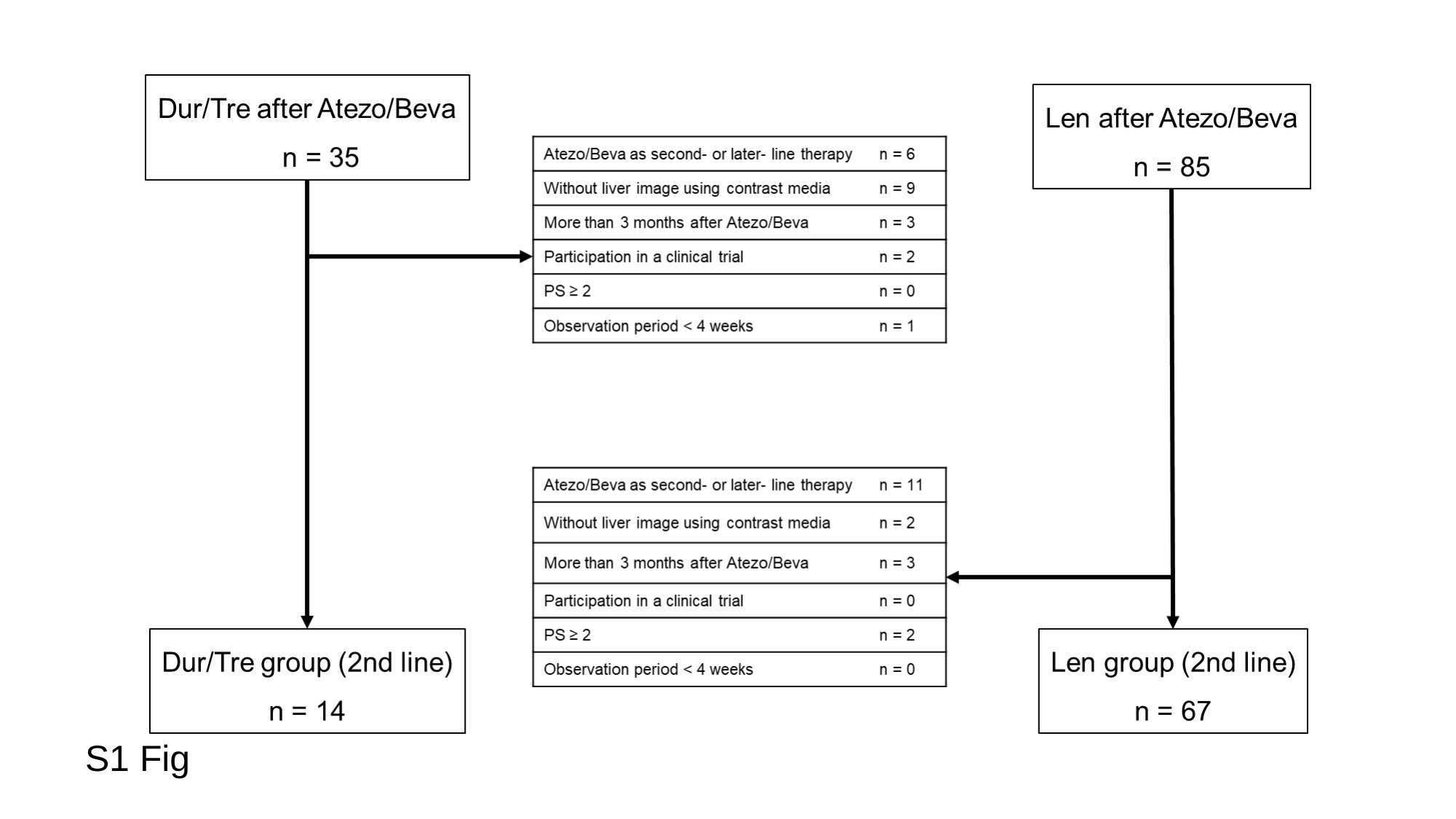

S1 Fig

Supplement: S1 Fig — Abbreviations: Dur/Tre, durvalumab plus tremelimumab; Len, lenvatinib; Atezo/Beva, atezolizumab plus bevacizumab; PS, performance status. (PPTX) [file pone.0341395.s001.pptx]

## Slide 1
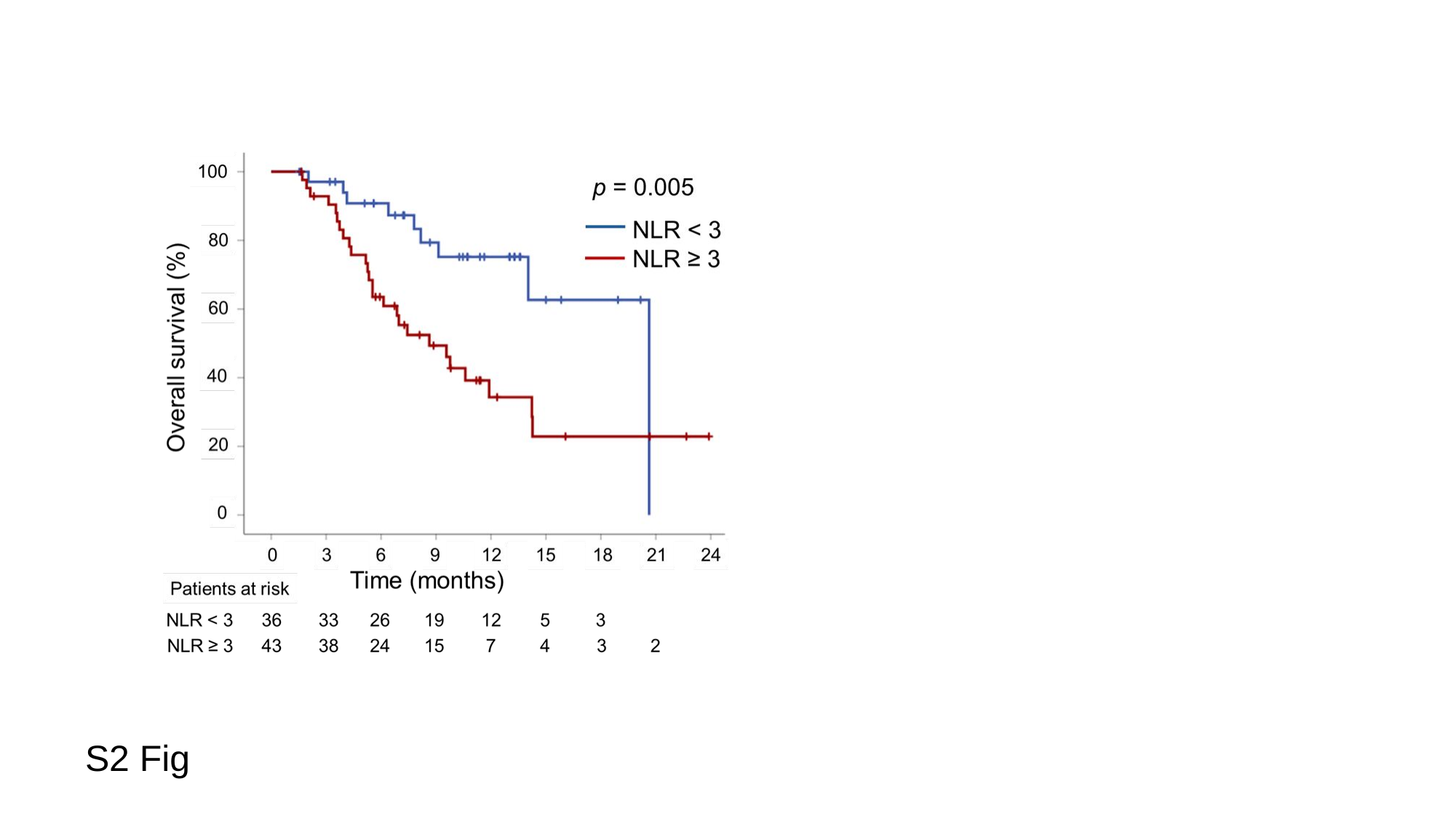

S2 Fig

Supplement: S2 Fig — Abbreviations: OS, overall survival; NLR, neutrophil-to-lymphocyte ratio. (PPTX) [file pone.0341395.s002.pptx]
