# Supplementary material for: Comparison of outcomes of second-line durvalumab plus tremelimumab versus lenvatinib following first-line atezolizumab plus bevacizumab in unresectable hepatocellular carcinoma
Source: PLoS One. 2026 May 7;21(5):e0341395. doi: 10.1371/journal.pone.0341395 (PMC13152131; doi:10.1371/journal.pone.0341395)
Supplement: S1 Table — Abbreviations: Dur/Tre, durvalumab plus tremelimumab; Len, lenvatinib; TACE, transcatheter arterial chemoembolization; HAIC, hepatic arterial infusion chemotherapy; PD, progressive disease; TAI, transcatheter arterial infusion; RT, radiation therapy. (DOCX) [file pone.0341395.s003.docx]

**S1 Table. Subsequent Therapy**

|  | Dur/Tre group  (n = 14) | Len group  (n = 66) |
| --- | --- | --- |
| Systemic treatment | 9 | 29 |
| Systemic treatment+TACE | 0 | 3 |
| Systemic treatment+HAIC | 0 | 1 |
| TACE | 1 | 5 |
| TACE+Continuation beyond PD | 0 | 1 |
| TAI/HAIC | 0 | 2 |
| RT | 0 | 5 |
| RT+Continuation beyond PD | 0 | 1 |
| Continuation beyond PD | 0 | 1 |
| Best supportive care | 4 | 18 |
| Abbreviations: Dur/Tre, durvalumab plus tremelimumab; Len, lenvatinib; TACE, transcatheter arterial chemoembolization; HAIC, hepatic arterial infusion chemotherapy; PD, progressive disease; TAI, transcatheter arterial infusion; RT, radiation therapy | | |
